# C G composition in transposon-derived genes is increased in FXD with perturbed immune system

**DOI:** 10.1093/narmme/ugae015

**Published:** 2024-10-10

**Authors:** Tamaki Suganuma, Huzaifa Hassan, Madelaine Gogol, Jerry L Workman

**Affiliations:** Stowers Institute for Medical Research, 1000 E. 50^th^ Street, Kansas City, MO 64110, USA; Stowers Institute for Medical Research, 1000 E. 50^th^ Street, Kansas City, MO 64110, USA; Stowers Institute for Medical Research, 1000 E. 50^th^ Street, Kansas City, MO 64110, USA; Stowers Institute for Medical Research, 1000 E. 50^th^ Street, Kansas City, MO 64110, USA

## Abstract

Increasing incidence of Fragile X disorders (FXD) and of immune-mediated disorders in FXD suggests that additional factors besides *FMR1* mutations contribute to the pathogenesis. Here, we discovered that the expression levels or splicing of specific transposon element (TE)-derived genes, regulating purine metabolism and immune responses against viral infections are altered in FXD. These genes include HLA genes clustered in chr6p21.3 and viral responsive genes in chr5q15. Remarkably, these TE-derived genes contain a low A T/C G suggesting base substitutions of A T to C G. The TE-derived genes with changed expression levels contained a higher content of 5′-CG-3′ dinucleotides in FXD compared to healthy donors. This resembles the genomes of some RNA viruses, which maintain high contents of CG dinucleotides to sustain their latent infection exploiting antiviral responses. Thus, past viral infections may have persisted as TEs, provoking immune-mediated disorders in FXD.

## Graphical Abstract

**Graphical Abstract:**
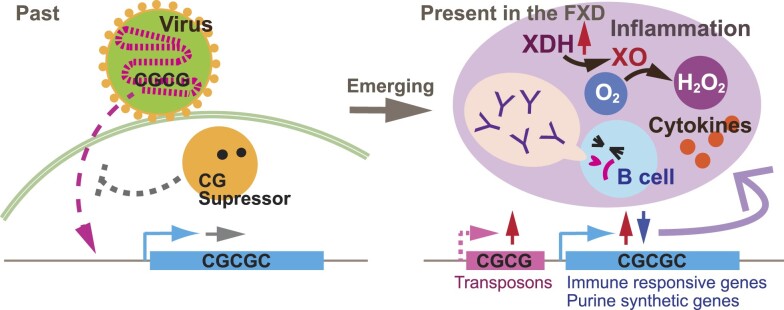

## Introduction

Fragile X syndrome (FXS) is an X-linked inherited intellectual disability caused by >200 CGG repeats mutation (‘full mutation’) in the fragile X messenger ribonucleoprotein 1 gene (*FMR1*) (1). The *FMR1* gene is also involved in Fragile X-associated tremor/ataxia syndrome (FXTAS) and Fragile X-associated primary ovarian insufficiency (FXPOI) carrying 55–200 CGG repeats (fragile X ‘premutation’). These three conditions are referred to Fragile X-associated disorders (FXD) (2). Please note, using the term ‘retardation’ in gene names has been noted to lead to undesirable influences in society, and alternative names have been adopted (3). Penetrance of FXS does not accommodate the X-linked dominant pattern (4). A large clinical cohort study of FXS patients showed an over-representation of infectious diseases, including otitis media, viral enteritis and candidiasis (5). Though autoimmune disorders have been underrepresented in FXS patients (5,6), the relative risk of immune-mediated disorders, such as rheumatoid arthritis, among females with heterozygotes of the premutation has been suggested (6). DNA alkylation damage causes O^6^meG:C and O^6^meG:T mismatches. These mismatches promote interactions of the mismatch repair (MMR) protein MSH6 with the molybdopterin synthase associating complex (MPTAC) and the Ada2a-containing histone acetyltransferase complex (ATAC) which promote sterol biosynthesis in response to alkylation damage. However, this function has been disrupted in lymphoblastoid cell lines (LCLs) from FXD patients (7). Importantly, in these FXD cells, the activity of xanthine oxidase (XO), which is converted from xanthine dehydrogenase (XDH) upon inflammation or low O2 tension (8), is elevated compared to the healthy donor cells (7). This indicates sustained inflammation in FXD patients (7). However, the causes of inflammation in FXD are unknown.

**Figure 1.**
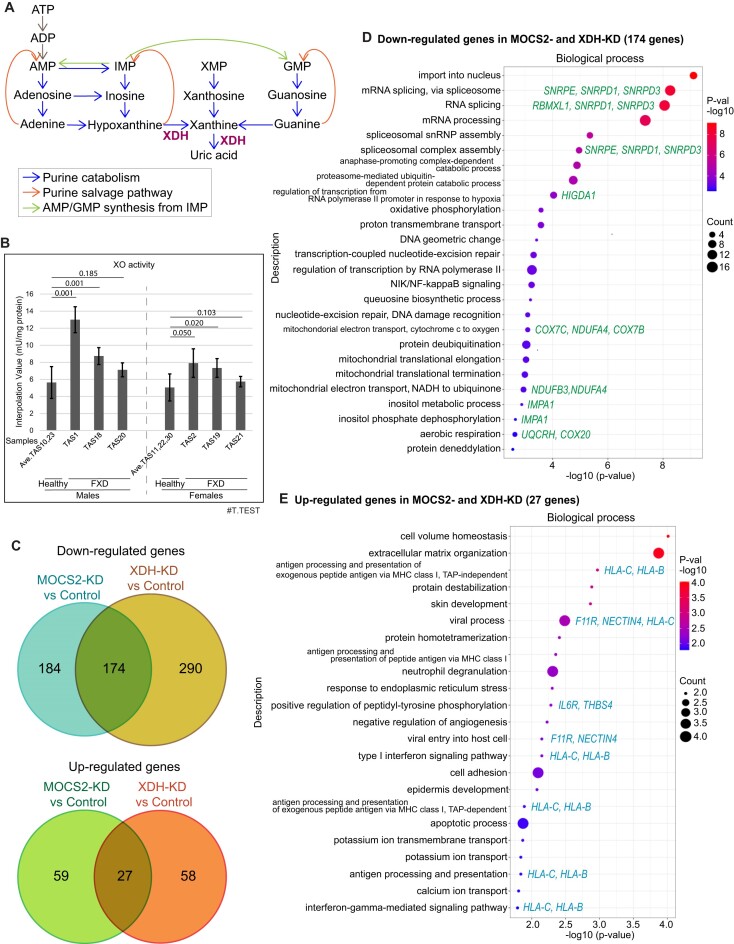
XDH and MOCS2 are essential for translations of genes regulating responses to viral infection and oxidative phosphorylation. (**A**) Purine catabolism. (**B**) Xanthine oxidase (XO) activity in cytoplasmic extracts of LCLs were measured by fluorometric assay (mean ± SD, *n* = 2). Averaged XO activities in healthy donor cells were compared with each FXD cells in each gender. The numbers of two-tailed test (T.TEST) are indicated. (**C**) Area-proportional venn diagrams indicating the numbers of genes with significantly changed transcript levels (adjusted *P*-value <0.05 and fold change >2) in polysomes in MOCS2- or XDH-knockdown cells compared to controls (*n* = 2 or 3). (D and E) Dot plots display GO analysis clustered by biological process of genes with decreased (**D**) or increased (**E**) transcripts in polysomes in MOCS2- and XDH-knockdown cells compared to polysomes in controls by RNA-seq. Representative genes are indicated.

Xanthine oxidoreductase (XOR) consists of four redox centers: a molybdenum center, a flavin adenine dinucleotide (FAD) center, and two 2Fe–2S clusters (8). XOR has two forms: XDH and XO (9). Purine hydroxylation occurs at the molybdenum center of XOR and requires the molybdenum cofactor (Moco) that is synthesized by Moco biosynthetic enzymes including MOCS2 (8,10). Interestingly, XDH is also essential for MOCS2 mRNA translation (11). Notably, MOCS2 is required for cellular levels of guanine and uridine and is essential for formation of the MPTAC which suppresses reactive oxygen species (12). Under normal O_2_ tension, XDH oxidizes hypoxanthine to xanthine and oxidizes xanthine to uric acid in the last step of purine catabolism (Figure 1A), and its 2Fe–2S clusters transfer electrons to the FAD where NAD^+^ is reduced to NADH. Under hypoxia or inflammation, XDH is converted to XO, which has a lower affinity to NAD^+^ than XDH, XO oxidizes xanthine to uric acid; however, electrons are instead transferred to molecular oxygen, generating superoxide and hydrogen peroxide as FAD levels are robustly reduced (8,10). This condition, in turn, reduces XO affinity for xanthine (10).

Numerous viruses reprogram their host cells to decrease mitochondrial respiratory rates (oxidative phosphorylation) in order to facilitate their replication (13). This reprogramming induces hypoxic responses, most of which are mediated by HIF1A (13). Hypoxia-induced pro-inflammatory cytokines promote expression of XDH (8). However, MOCS2 is essential for HIF1A translation (14). Therefore, there is an axis between HIF1A expression, XDH activity, and MOCS2 expression. Retroviruses and retrotransposons share a common structural organization (15). Retroviruses and the long terminal repeat (LTR) retrotransposon have a purine-rich RNA segments, the polypurine tract (PPT), where the second strand or plus-strand DNA synthesis is initiated (16,17). The difference is that the retrotransposons have lost the envelope genes of retroviruses (15). In this study, we hypothesized that virus-like stimuli might trigger perpetual inflammation in FXD. We asked what elevated XO activity suggests about the pathogenesis of FXD, and whether expressions of transposons are related to perpetual inflammation in FXD.

## Materials and methods

### Examination of knockdown HEK293 cells

HEK293 cells transfected with target gene-specific CRISPR-Cas9 expression clones were cultured in DMEM-High Glucose (4500 mg/l) (D6429, SigmaAldrich, Missouri, USA) with 10% fetal bovine serum and 500 μg/ml Geneticin (Life Technologies) at 37°C for 21 days. The cells were lysed in a 5× volume of ice-cold Extraction Buffer I (10 mM 4-(2-hydroxyethyl)-1-piperazine ethanesulfonic acid (pH 7.5), 1.5 mM magnesium chloride, 10 mM potassium chloride, 1 mM dithiothreitol and proteinase inhibitors) for 10 min, followed by incubation with 1% nonoxynol-40 for 5 min. Cytoplasmic extracts were obtained from supernatants after centrifugation at 2655 g for 5 min (18) and were examined by western blotting with antibodies against MOCS2 (sc-377169, Santa Cruz), xanthine dehydrogenase/oxidase (35792, Cell Signaling Technology), eIF4E (sc-9976, Santa Cruz), TRL3 (83136–3-RR, proteintech), IRF-5 (sc-56714, Santa Cruz), TSC1 (6935, Cell Signaling Technology), P4HA2 (66604-1-Ig, proteintech), CD46 (sc-52647, Santa Cruz), NDUFS3 (sc-374282, Santa Cruz), THOC1 (sc-514123, Santa Cruz), PLSCR1 (11582-1-AP, proteintech), DDX17 (sc-398168, Santa Cruz) and eIF3β (sc-374156, Santa Cruz, RRID:AB_11008477).

### Target gene-specific CRISPR-Cas9 expression clones (GeneCopoeia)

-3 × sgRNA/Cas9 all-in-one expression clones targeting:

MOCS2 (NM_176806.2) (HCP211287-CG01-3)

XDH (NM_000379.3) (HCP218552-CG01-3)

LOC84661 (NM_032574.2) (HCP220858-CG01-3)

### Culture of human lymphoblastoid cell lines (LCLs)

LCLs from B-lymphocytes from FXS and apparently healthy donors listed in Supplementary Table 2 were provided by the Coriell Institute for Medical Research and were cultured in RPMI1640 containing 2 mM l-glutamine and 15% fetal bovine serum at 37°C under 5% CO_2_. Details are described in this site: https://www.coriell.org/0/Sections/BrowseCatalog/DiseaseDetail.aspx?PgId=403&omim=MEN30955&coll=

### Polysome assay

Isolation and sedimentation of polysomes were performed as described previously (19) with modifications. Briefly, ∼1.5e7 cells were cultured in growth media containing 100 μg/ml cycloheximide for 5 min at 37°C and were washed twice with PBS containing 100 μg/ml cycloheximide. The collected cells were incubated with a 7× volume of hypotonic buffer [5 mM Tris–HCl (pH 7.5), 2.5 mM MgCl_2_, 1.5 mM KCl, and protease inhibitor cocktail (EDTA-free), 100 μg/ml cycloheximide, 2.35 mM DTT, and 0.235 U/ml RNase inhibitor] based on the volume of the cell pellet and were mixed with 0.5% TritonX-100 and 0.3525% sodium deoxycholate. After vortexing for 10 s, the cell suspensions were incubated on ice for 15 min. Lysates were taken from the supernatants after centrifugation at 16 000 g for 7 min. OD_260_ for each lysate were adjusted to 100–125 OD_260_ units. 5 OD_260_ units of each lysate was kept as inputs. 460 μl of each sample was loaded onto each 5–50% sucrose gradient and was centrifugated at 36 000 rpm for 2 h at 4 °C using a SW41Ti rotor. Each gradient was fractionated by the fraction collector, and the fraction profile was recorded. The total RNA in fractionated lysates and input was purified using TRIzol reagent (ThermoFisher, Cat# 15596026) and Direct-zol RNA MiniPrep Plus (Zymo Research, R2072).

### Xanthine oxidase fluorometric assay

Cytoplasmic extracts were analyzed using a Xanthine Oxidase Fluorometric Assay Kit (Cayman, 10010895). Samples were diluted at a 1:3 ratio using sample buffer (10 mM 4-(2-hydroxyethyl)-1-piperazine ethanesulfonic acid pH 7.5, 10 mM potassium chloride, 1.5 mM magnesium chloride and one Roche Complete EDTA-free protease inhibitor tablet per 15 ml buffer). Both initial and subsequent dilutions of the standard were done per protocol using sample buffer described above. In addition, the assay was miniaturized to 384 well format with a final well volume of 25 μl by reducing each component of the protocol by a factor of 4. The assay measured the production of the fluorescent compound resorufin (excitation 520–550 nm, emission 585–595 nm), produced by the reaction of H_2_O_2_ with 10-acetyl-3,7-dihydroxyphenoxiazine (ADHP) in a 1:1 stoichiometry in the presence of horseradish peroxidase. Absorbance was measured at 90 min after XO substrate was added to the sample mixtures. The average background (sample buffer only, *n* = 3) was subtracted from all sample and standard curve values. The standard curve was plotted as a linear regression and the interpolation of the sample's values determined the XO concentration (μU/ml). The amount of XO for each sample was normalized to protein concentration and was plotted as mean ± SD. Each sample contained three technical replicates, and the experiment assayed two biological replicates of each sample.

### Assay for citrate synthase activity

Isolation of mitochondria in LCLs was performed as described previously (20). Cell pellets derived from 1e7 cells were incubated with extraction buffer [0.25 M sucrose, 20 mM HEPES–KOH (pH 7.5), 10 mM KCl, 1.5 mM MgCl_2_, 1 mM EDTA, 1 mM EGTA, 1 mM DTT and 0.1 mM PMSF. The cells were homogenised with a Teflon-glass homogenizer and were centrifuged at 750 g for 10 minutes to obtain mitochondrial extracts from the supernatant. Citrate synthase activity of 1/50 times diluted mitochondria extracts was examined by MitoCheck® Citrate Synthase Activity Assay Kit (704010, Cayman), and absorbance at 412 nm with 30 second kinetic interval for 20 min was measured by SpectraMax iD3 (Molecular Devices).

### RNA sequencing

For total RNA from polysomes in HEK293 cells, ribo-depleted, stranded 50-base single-end reads were generated on a Hiseq 2500. Reads were demultiplexed allowing up to one mismatch using Illumina bcl2fastq2 v2.18. Reads were aligned to UCSC genome hg38 using TopHat version 2.0.13, with –library-type fr-firststrand, using gene models from Ensembl 91. Read counts on genes were generated using HTSeq-count with -m intersection-nonempty.

For short read RNA sequencing, total RNA from LCLs was ribo-depleted and stranded 100 base single-end reads were generated on a NextSeq 2000 (*n* = 3). RNA-seq reads were demultiplexed into fastq format allowing up to one mismatch using Illumina Illumina's bcl2fastq2 (v 2.20). Subsequently, the reads were aligned to *hg38* reference genome from University of California at Santa Cruz (UCSC) using STAR (v 2.7.3a). The gene model retrieved from Ensembl, release 102 was used to generate gene read counts.

For long read RNA sequencing, total RNA was used for RNA library preparation for each synthetic poly-A tails (*n* = 2) and normal RNA library (*n* = 2). The RNA was directly sequenced as single read on the PacBio RS Sequel- II-3000 provided by Cold Spring Harbor Laboratory. The reads from each sample were aligned to the human genome (hg38).

### Statistical information

For analysis of transcripts from polysome fractions in HEK293 cells using Illumina-RNA-seq data, read counts on genes were generated using HTSeq-count with -m intersection-nonempty. Downstream analysis was done in R (3.5.0). Venn diagrams were generated using Vennerable (3.0). Differential expression using Illumina RNA-seq data was done using edgeR.

For differential expression analysis using short read RNA sequencing (Illumina) of ribo-depleted RNA from LCLs, the gene model retrieved from Ensembl, release 102 was used to generate gene read counts. The transcript abundance TPM (Transcript per Million) was quantified using RSEM (v 1.3.0). Differentially expressed genes were determined using the R package edgeR (v 3.36.0) after filtering low-expressed genes with a ‘CPM’ (counts per million) of 0.5 in at least one library. The resulting P-values were adjusted with the Benjamini–Hochberg method using R function p.adjust. Genes with an adjusted *p-value* less than 0.05 and a fold change of at least two were considered as differentially expressed. Gene functional enrichment analysis for LCLs was performed using a custom script built over R package ‘clusterProfiler’ (v 4.4.4). *hg38* Gene-GO terms retrieved from Ensembl BioMart were used to identify over-represented GO terms in different gene sets. To identify the presence of Transposable Element's, the Transposable elements (TE) consensus sequences from Dfam database are aligned with all the samples using pbmm2 with –preset ISOSEQ and –best-n 10 (To capture up-to 10 multiple alignments).

To analyze base substitutions from Illumina RNA-seq data, consensus sequences for each gene were generated from the RNA-seq reads using samtools consensus (v1.18). Bases with a minimum fraction of 0.51 were referred to as consensus, and if no consensus was reached, the base was labeled as ‘N.’ Failed bases (N) were included in the analysis, but no insertions or deletions were considered. Base substitutions were determined by comparing the consensus sequence of each gene with the reference sequence. The total base substitutions for each gene were normalized to the gene length per 1000 bp. The normalized values are displayed in boxplots using log2 scaling.

For A T > C G substitutions, the total includes A > C, A > G, T > C and T > G base substitutions; for C G > A T substitutions, the total includes C > A, C > T, G > A, and G > T base substitutions. The ratio of A T > C G to C G > A T was then calculated, and the average ratio for each sample across replicates is shown in the heatmap.

**Figure 2.**
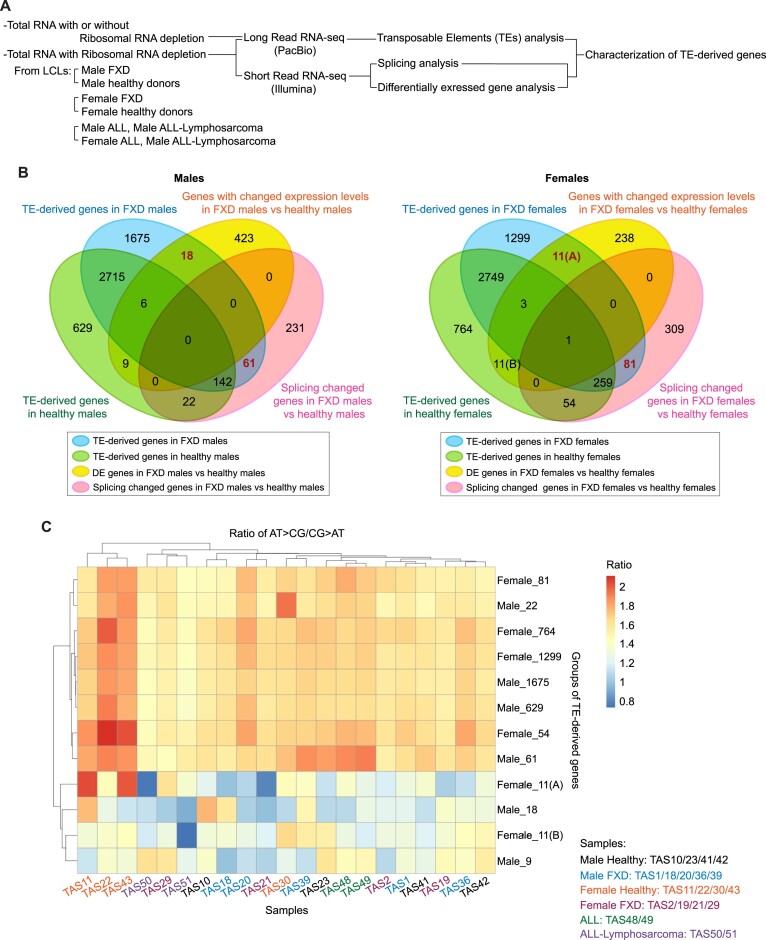
Low A T / C G leads to altered expressions of TE-derived genes in FXD. (**A**) Diagram of the RNA-seq strategy used in this study. (**B**) Venn diagram shows transposable elements (TEs)-derived genes identified using PacBio-reads and overlapped with splicing changed genes and genes with changed expression levels (differentially expressed (DE) genes) identified using Illumina-short reads, in males (left) and females (right). (A) and (B) are indicated to distinguish between each group of 11 genes for convenience. (**C**) Heatmap with rows for each group of genes corresponding to those in (B) and columns for samples displays the raw data of ratio of AT > CG / CG > AT, which were analyzed by the base substitutions of each nucleotide (A T > C G: sum of A > C, A > G, T > C and T > G base substitutions; C G > A T: sum of C > A, C > T, G > A and G > T base substitutions), in averaged sum (*n* = 3) of the base substitutions.

For CpG island analysis, the CpG islands for the hg38 genome were obtained by using the UCSC CpG island track from UCSC. The CpG islands were intersected with the genes from the Venn diagram shown in Figure 2B for specific sections and any CpG islands that overlapped with the genes with at least 1 bp were taken. The location of CpG islands in the gene body ± 2kb flanking regions was plotted. The plots have been smoothed using UnivariateSpline from scipy starting with 5 as the smoothing factor.

PacBio long reads were analyzed using Iso-Seq (v 3) starting with the generation of consensus sequences from subreads. Subsequently, poly-A tails were trimmed, and the resulting full-length isoforms were then clustered to generate unpolished isoforms, which were further polished using subreads. The polished isoforms were aligned to the hg38 reference genome using pbmm2 (v 1.7.0) and collapsed based on genomic alignments.

The transcripts generated from Iso-Seq were characterized using SQANTI (v3.4.2) with gene models retrieved from Ensembl release 102. Unannotated isoforms, isoforms mapped to antisense strand of an annotated gene, fusion genes and novel genes were removed for downstream analysis.

## Results and discussion

### MOCS2 and XDH translationally regulate response to viral infections

Since XO activities are increased in FXD (Figure 1B) (7), we wondered whether roles of XDH in translation and purine catabolism are related to the pathogenesis of FXD. MOCS2, required for the synthesis of the molybdopterin cofactor of XHD, is also essential for translation (11); therefore, we examined RNA-sequencing of total RNA from extracts (the inputs for polysome fractionation; ‘In’) or from polysomes (≥3; ‘Po’) in MOCS2-, XDH-, and control-knockdown HEK293 cells (Supplementary Figures S1A and B) (11). We found that transcripts of 174 genes were significantly decreased and transcripts of 27 genes were increased in polysomes from MOCS2- and XDH-knockdown cells compared to controls (Figure 1C). Genes regulating mitochondrial electron transport, such as *NDUFB3*, *NDUFA4* and *COX7C*, and the hypoxia response, such as *HIGD1A*, were downregulated in MOCS2- and XDH-knockdown cells (Figure 1D, Supplementary Table S1A). The transcripts of splicing factors, which are subunits of MPTAC, such as SNRPD1, -3 (12), were significantly reduced in MOCS2- and XDH-knockdown cells (Figure 1D). By contrast, transcripts of genes involved in viral entry into the host cells or anti-viral processes, such as *F11R*, *NECTIN4*, and *HLA-B*, were significantly increased in polysomes from MOCS2- and XDH-knockdown cells (Figure 1E, Supplementary Table S1B). Most genes with increased transcripts in polysomes from XDH-knockdown were associated with viral defense systems, immune responses, and hypoxia response, including RSAD2 (21) (Supplementary Figure S1D, Supplementary Table S1C). MOCS2 transcripts were significantly reduced in polysome of XDH-knockdown cells (Supplementary Figure S1E, Supplementary Table S1D). Thus, MOCS2 and XDH are critical for mitochondrial electron transport, utilization of molecular oxygen to generate ATP, and splicing. MOCS2- and XDH-knockdown induce translation of genes responding to hypoxia and of genes defending viral infections, suggesting that functions of MOCS2 and XDH may accommodate the process of viral infection.

### Splicing or expression level of TE-derived genes is changed in FXD compared to healthy donors

We next searched for the potential source of increased XO activities and inflammation in FXD patients. As retroviruses and retrotransposons share common features of sequence structure (15), we examined whether expression of transposons was changed in FXD compared to healthy donors. We examined the total RNA and mRNA (polyA selection) of Lymphoblastoid Cell Lines (LCLs) from FXD patients and healthy donors by long-read RNA sequencing (PacBio) (Figure 2A, Supplementary Table S2). The reads were aligned to annotated transposable element (TE) DNA sequences using Dfam database (22). We searched annotated TE-derived genes in individual cells and then merged them within FXD cells and healthy donor cells separately (Figure 2A). We also examined the expression levels of all the genes by ribo-depleted short-read single stranded RNA-seq (Illumina) (Figure 2A). We then compared the genes with significantly different expression levels in comparisons between FXD cells and healthy donor cells in each gender (*P*-values were corrected using FDR method and genes at less than 5% FDR and with 1 > a log_2_(fold change) < –1 for up and down-regulated genes were counted as genes with changed expression levels). Since TE expression often interrupts splicing (23), we examined splicing using rMATS (v 4.1.1) (24) by short-read RNA-seq and compared the genes with altered splicing between healthy donor cells vs FXD cells in each gender through comparison with annotated splicing (Figure 2A). We found that expression levels of 18 TE-derived genes were changed in male FXD vs male healthy donors (Figure 2B). Expression levels of 11 TE-derived genes were changed in female FXD vs female healthy donors (Figure 2B, ‘11(A)’). Correspondingly, compared to FXD, expression levels of 9 TE-derived genes were changed in male healthy donors (Figure 2B, ‘9’) and expression levels of 11 TE-derived genes were changed in female healthy donors (Figure 2B, ‘11(B)’). Splicing of 61 TE-derived genes detected in only male FXD were altered in male FXD compared to male healthy donors and of 81 TE-derived genes detected in only female FXD were changed in females FXD compared to female healthy donors (Figure 2B). Compared to FXD, splicing of 22 TE-derived genes were changed in male healthy donors, and splicing of 54 TE-derived genes were changed in female healthy donors (Figure 2B). The splicing changed TE-derived genes and TE-derived genes with changed expression levels did not overlap in males and were mostly not overlap in females (Figure 2B).

### Substitutions of A T to C G alter the expressions of TE-derived genes in FXD

XDH is required for purine catabolism; however, the affinity of XO for xanthine is reduced under hypoxia (10) (Figure 1A). In addition, MOCS2 is essential for cellular levels of guanine (8,12,25). Hypoxia, which favors many viruses, stimulates purine synthetic enzymes but restricts oxidative phosphorylation (13,26,27). Therefore, we next asked whether purine metabolism is involved in changes in the expressions of TE-derived genes in FXD. Chargaff's second rule states A% ≈ T% and G% ≈ C% are valid for individual strands of the two DNA strands (28). However, there are substantial local differences of base composition due to asymmetry of A versus T and G versus C rather than A T versus G C on the codon-synonymous strand (29). The asymmetries are present in the genomes of SV40 and polyoma viruses in locations corresponding to transcriptional boundaries and purine-rich on the codon-synonymous strand (29). We analyzed the nucleotide composition of TE-derived genes with changed expressions (splicing or expression levels) in FXD. To obtain a notion of the relevance of previous viral infections with changed expressions of TE-derived genes, we also analyzed the nucleotide composition by RNA-seq (Illumina) for total RNA in LCLs from patients diagnosed with acute lymphocytic leukemia (ALL) (TAS48 and TAS 49) and ALL-Lymphosarcoma (TAS50 and TAS51), which are possibly transformed with Human T-cell Leukemia Virus (HTLV), because, adult T-cell leukemia/lymphoma occurs in approximately 5% of HTLV-1 infected individuals (30,31) (Supplementary Table S2). We generated a consensus sequence for each gene from RNA-seq reads (Illumina) using samtools consensus. The consensus sequence of each gene was compared to the reference sequence and then base substitutions were calculated (29). At least 0.51 fraction of bases was referred as consensus to emit that base type. The failed base was referred to ‘N’, including No insertions or deletions. All the base substitutions were summed, and the sum was normalized to length of the gene in KB (sum of all base substitutions / (length of the gene/ 1000 bp)). We took the ratio of A T > C G / C G > A T and then analyzed the base substitutions of each nucleotide (A T > C G: sum of A > C, A > G, T > C and T > G base substitutions; C G > A T: sum of C > A, C > T, G > A and G > T base substitutions). We averaged the replicates (average ratio, n = 3) of each sample and generated a heatmap with rows as each group of genes corresponding to the numbers in Figure 2B and columns as samples (Figure 2C). The Z-scores were illustrated with a heatmap to further illustrate the different ratios in each sample relative to the average (Supplementary Figure S2). We found that in most of samples regardless of FXD and healthy donors the ratio of A T > C G / C G > A T in group male 18, group female 11(A), group male 9, and group female 11(B) (groups with changed expression levels in FXD or healthy donors shown in Figure 2B) were lower than that in the other groups (Figure 2C). In these groups, A T > C G / C G > A T in female FXD (TAS2/19/21/29), ALL (TAS48/49) and ALL-Lymphosarcoma (TAS50/51) were particularly lower than other samples (Figure 2C; Supplementary Figure S2). Interestingly, A T > C G / C G > A T in almost all groups of TE-derived genes were lower in female FXD (TAS2/19/21/29) and both genders of ALL-Lymphosarcoma (TAS50/51) compared to those in female healthy donors (TAS11/22/30/43) although there were variations among healthy donors in group female 11(A) (Figure 2C; Supplementary Figure S2). Especially, in groups of male 61, male 22, female 81 and female 54 (changed splicing in FXD or healthy donors), A T > C G / C G > A T in female FXD (TAS2/19/21/29) were lower than female healthy donors as seen in the Z-score analysis (Supplementary Figure S2). Thus, substitutions of A T to C G frequently occurred in TE-derived genes with changed expression levels or changed splicing in female FXD compared to healthy female donors. Decreases in A T > C G / C G > A T in specific TE-derived genes, which led to alterations of the expression in FXD, were also observed in ALL and ALL-Lymphosarcoma (Figure 2C; Supplementary Figure S2). Hereafter we use nomenclature substitutions of ‘A T to C G’ as we studied the frequency of A T: C G.

### High content of CG dinucleotides changes the expression levels of TE-derived genes

**Figure 3.**
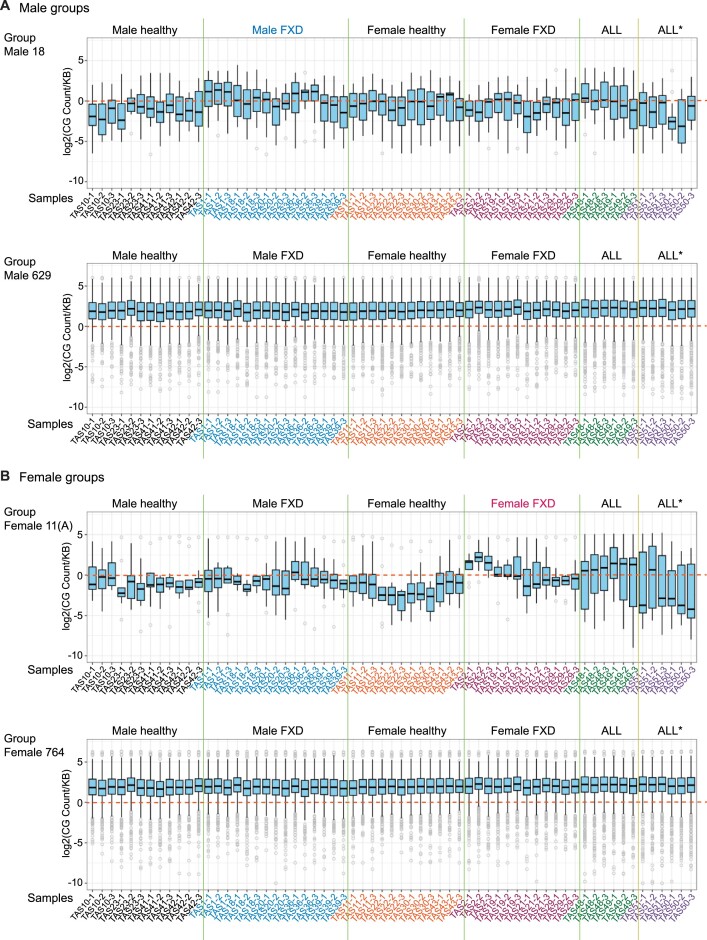
5′-CG-3′ are increased in TE-derived genes with altered expressions in FXD. (**A**, **B**) Box plots display the count of CG di-nucleotides normalized to length of the gene in 1000 bp using the ATGC consensus in each group of TE-derived genes corresponding to the groups of Venn diagram in Figure 2B (Supplementary Figures S3 and S4). Sample names and repeats are indicated at the bottom. The count in each repeat (*n* = 3) is indicated. ALL*: ALL-lymphosarcoma.

Vertebrate genomes contain lower 5′-CG-3′dinucleotides than expected number (CG suppression) (32). It has been proposed that a host antiviral protein, ZC3HAV1/ZAP, exploits host CG suppression to identify viral RNA (non-self RNA) through binding CG-enriched elements of the HIV-1 genome (32). ZC3HAV1/ZAP also suppresses the transcription of delta-type retroviruses, including HTLV-1 and simian T-cell leukemia virus (STLV), via its binding to CG dinucleotides (30,32). However, genomes of many RNA viruses mimic the CG suppression of their hosts’ genome (33–35) for their replication (32). Interestingly, it has been suggested that HTLV-1 takes advantage of antiviral systems, like ZAP, by maintaining high contents of CG dinucleotide to reduce viral antigen expression, leading to sustainment of latent infection (30). We therefore measured CG dinucleotide via generating consensus sequence of each gene using RNA-seq reads as described above (Figure 2C). The CG count was normalized to length of the gene in KB (CG count/(length of the gene/1000)). We compared CG count/kb in TE-derived genes of each sample between groups. We found that CG levels in TE-derived genes with changed expression levels (groups 18 and 9 in males, groups 11(A) and 11(B) in females, Figure 2B) were mostly below zero and were lower than unchanged TE-derived genes (groups 629 and 1675 in males, groups 764 and 1299 in females, Figure 2B) (Figure 3A and B; Supplementary Figures S3 and S4). In group male 18, CG levels in male FXD were higher compared to those in male healthy donors (Figure 3A). In group female 11(A), CG levels in female FXD were also higher than those in female healthy donors (Figure 3B). Therefore, in groups 18 and 11(A) CG levels were likely suppressed in healthy donors; however, they were increased in FXD in each gender (Figures 3A and 3B). In addition, CG levels in ALL were higher than healthy donors in these groups (Figure 3A and 3B). We were unable to find the reasons that CG levels in a male healthy (TAS10) were exceptional in male healthy group 9 (Supplementary Figure S3). Together, these data suggested that increased substitutions of A T to C G and increased CG dinucleotides lead to changes in the expression levels of TE-derived genes in FXD. This may be similar to substitutions that prolonged latent infection of HLTV-1 in ALL.

Notably, ‘5′-GC-3′’ dinucleotides levels in male group 18 and in female group 11 (A) were also increased in FXD and ALL compared to healthy donors in each gender (Supplementary Figures S5 and S6). GC dinucleotide levels throughout the groups were higher than CG dinucleotide levels (Supplementary Figures S3, S4, S5 and S6). In *Escherichia coli*, AT to GC mutations were shown to be decreased in deficiencies of MMR including MutSα, MutL, and MutH (36). MutSα deficiency cells do not attempts to O^6^meG:C and O^6^meG:T mismatches in mammals (37). We previously found that DNA alkylation damage signaling was impaired by destabilized MutSα in FXD (7). Therefore, dysregulation of MMR might also influence the alteration of A T C G composition in FXD.

We also analyzed frequency of CpG islands in these TE-derived genes using UCSC CpG island track although it has been known that CpG islands are highly enriched in *Alu* repetitive elements of TEs (38). Interestingly, populations of CpG islands containing genes in male 18 group were relatively lower than those in male 61 and 1675 groups (Supplementary Figure S7A). This trend was also revealed in female 11 (A) group (Supplementary Figure S7A). The locations of these CpG islands enriched in the gene body, which may affect the gene expression if they are methylated, were relatively comparable among these male groups (Supplementary Figures S7B and S7C). These data suggested some mechanisms on changed expression levels in group 18 beside of regulation of CpG.

### Splicing of TE-derived genes regulating purine metabolism and immune responses is changed in FXD

**Figure 4.**
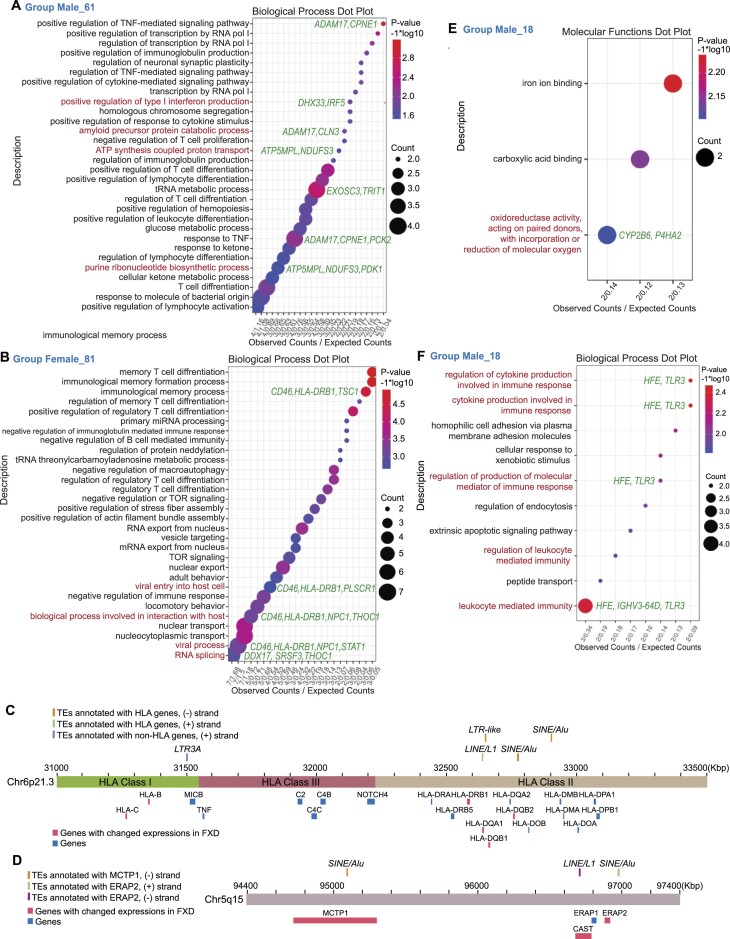
Expressions of TE-derived genes regulating purine metabolism and immune responses were changed in FXD. (A, B, E, F) Dot plots display GO analysis clustered by biological process (**A, B, F**) or molecular functions (**E**) for TE-derived genes with changed splicing corresponding to group male 61 (A) or group female 81 (B) in Figure 2B (*P* < 0.05) or changed transcript levels corresponding to group male 18 (E and F) in Figure 2B (adjusted *P* < 0.05) by using RNA-seq. The TOP 30 processes are listed in A and B. Representative genes are indicated. (C, D) Locations of HLA genes (**C**) and *ERAP2* (**D**) with/without changed expressions and TEs (Alu, LTR, LINE, and SINE) are illustrated.

To further understand the effects of alterations of TE-derived genes in FXD, we performed GO analysis for TE-derived genes with changed expressions in FXD compared to healthy donor (groups 18, 61 in male, groups 81 in female, Figure 2B). We found that splicing of the TE-derived genes regulating purine biosynthesis and ATP biosynthetic process including *ATP5MPL* and *NDUFS3* are changed in male FXD compared to male healthy donors (Figure 4A, Supplementary Table S3). NDUFS3 functions within mitochondrial complex I, and transcript levels of NDUFB3 and NDUFA4, subunits of mitochondrial complex I, were also reduced in polysomes of MOCS2- and XDH-knockdown cells (Figure 1D, Supplementary Table S1A). Therefore, we examined mitochondrial function by measuring citrate synthase activity in the LCLs. The citrate activities in FXD (TAS20/39/21) were clearly lower than healthy donors in each gender although there were variations of the activity in FXD cells (Supplementary Figure S8). Impairment of mitochondrial electron transport system and increased mitochondrial oxidative stress induces inflammation (39), suggesting inflammation in FXD. Splicing of genes involved in amyloid precursor protein metabolic process, such as *ADAM17* and *CLN3*, were also changed in male FXD (40) (Figure 4A). In female FXD, splicing of TE-derived genes associated with viral entry to host cells (*CD46*, *HLA-DRB1*, *NPC1*, and *PLSCR1*) (41), defense response to virus (such as *DDX17*, *PLSCR1*, and *STAT1*) (42,43), HLA gene (*HLA-DRB1*), and histone demethylation (*KDM6A*/*UTX* and *JMJD1C*) were changed (Figure 4B; Supplementary Tables S3, S4, and S5). By monitoring individual samples, splicing of some HLA genes located in chr6p21.3, including *HLA-DMA* and *HLA-DQB1*, and *HLA-*A and *HLA-C*, were changed in TAS1 male FXD, and TAS2 and 19 female FXD, respectively (Figure 4C; Supplementary Figure S9; Supplementary Tables S3 and S4). An Alu cluster, a LINE cluster, and purine-rich sequence have been found in chr6p21.3, which is a region between the Class II and III of the MHC (44) (Figure 4C). Alu elements accumulate mutations that activate cryptic splice sites, or polyadenylation sites, within the Alu (45). This can cause alternative splicing of RNA or premature termination of translation of genes into which they are inserted or nearby genes (45). Altered expression or splicing of HLA genes may have several important implications. Variations of three amino acids in HLA-DRB1 and two additional amino acids positions in HLA-B and HLA-DP, located in antigen peptide binding sites, confer rheumatoid arthritis (RA) susceptibility (46). HLA-DRB1 residues 71 and 74 are hotspots for other autoimmune disease-associated polymorphisms (47). HLA class II molecules influence susceptibility to inflammation (48). Thus, alteration of TE-derived genes might provoke immune-mediated disorders in FXD.

Notably, we found that splicing of *ERAP2* (chr5q15) was changed in TAS1, 18 and 20 male FXD versus male healthy donors (Figure 4D; Supplementary Figure S10; Supplementary Table S4). ERAP2 is a zinc metalloaminopeptidase, generating optimal-length peptides for loading onto MHC class I (49). ERAP2 reduces susceptibility to HIV infection (49). Influenza infection changes transcription and splicing of *ERAP2* loci, resulting in expressions of multiple variants of *ERAP2* (50). We found that in chr5q15 splicing of *MCTP1* in male FXD and splicing of *CAST* in female FXD were also changed compared to healthy donors in each gender (Figure 4D; Supplementary Tables S3–S5). MCTP1 has been shown to suppress oxidative stress response from glutamate toxicity and is related to vulnerability to neuropsychiatric diseases including bipolar disorder (51). *CAST* encodes calpain inhibitor (52). Calpain-mediated proteolysis modulates the phenotype of spinocerebellar ataxia type 3, which is caused by a CAG repeat expansion at *ATXN3* (52). Hence, disruptions of these genes in chr5q15 by TEs occurred in FXD may be beneficial to infection of some viruses, such as HIV.

### Expression levels of TE-derived genes regulating molecular oxygen are changed in FXD

**Figure 5.**
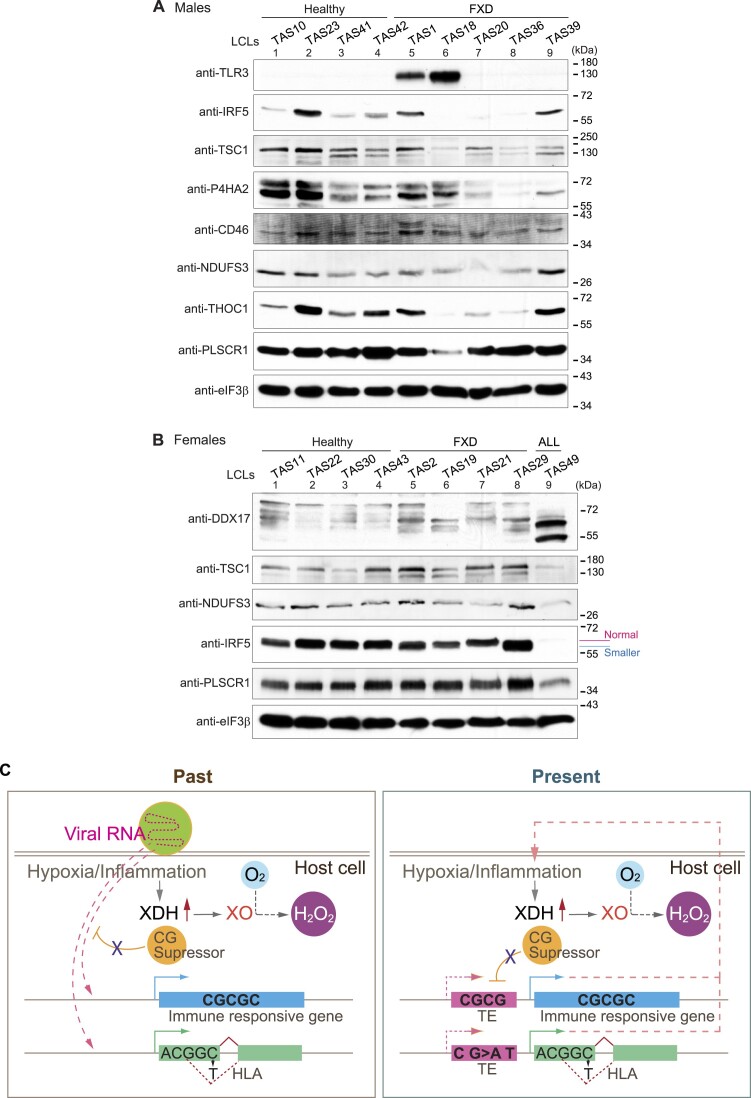
Protein expressions of TE-derived genes responding viral infection were changed in FXD. **(A, B)** Protein expressions of TE-derived genes regulating immune responses (TLR3, IRF5, TSC1, CD46 and THOC1), viral responses (PLSCR1 and DXX17), oxidoreductase activity (P4HA2), and mitochondrial function and purine metabolism (NDUFS3) in the extracts from indicated LCLs were analyzed by western blots. eIF3β was used as the loading control. (**C**) Summary model is illustrated. Viral RNA with a high content of CG dinucleotides is integrated into the host genome by exploiting CG suppressor to decrease antigens and is retained as retrotransposons (TEs) (left panel). Some viral infections trigger hypoxia, which promotes conversion of O_2_ to H_2_O_2_ via XO (left panel). Inflammation induces XDH expression and conversion of XDH to XO, producing ROS including H_2_O_2_ (left and right panels). In FXD in the present, increased substitution of A T to C G in TE-derived genes causes splicing changes, including in HLA genes (right panel). Increased CG dinucleotide changes expression of TE-derived genes regulating immune responses, suggesting that these genes had deceived CG suppression. Thus, maintaining high levels of C G content and CG dinucleotides in TE-derived genes likely stimulates XO activity and has maintained expression of TEs.

We found that the expression levels of TE-derived genes, *CYP2B6* and *P4HA2*, involving oxidoreductase activity were changed in male FXD (Figure 4E, Supplementary Table S6). Limited oxygen availability suppresses the activities of several JmjC demethylases including KDM6A, of which splicing was changed in female FXD (Supplementary Table S3), as their catalytic reactions require molecular oxygen (53). Furthermore, the expressions of TE-derived genes involving immune response, such as *TLR3*, were changed in male FXD (Figure 4F, Supplementary Table S6). We further examined the protein expression of changed TE-derived genes. In addition to changed protein levels of TRL3 and P4HA2 in FXD, the protein levels of IRF5 and NDUFS3, which regulates immune system and purine metabolism respectively, were also altered in some FXD cells compared to healthy donor cells in each gender (Figure 5A and B). Protein levels of DDX17, which acts in the defence response to viruses (Figure 4B; Supplementary Table S3), were changed in TAS19/29 female FXD and TAS49 female ALL (Figure 5B). Previous studies have shown that nitrogen oxide and superoxide are generated by alveolar phagocytic cells upon influenza infection (54). Low O_2_ tensions also favors Hepatitis C virus replication (26). Hence, roles of TE-derived genes with changed expressions in FXD deal with molecular oxygen. Low oxygen tension can be favorable for viral infection and readily generates reactive oxygen species (ROS). Remember that increased XO activity seen in FXD (7) generates ROS from oxygen (55,56). Notably, TE-derived genes regulating molecular oxygen signals and the immune system have been changed together in FXD. Yet, we were unable to dissect the reasons for gender-based differences in the expressions of TE-derived genes in FXD.

Altogether, we found that substitutions of A T to C G including increased CG dinucleotides changed expression of TE-derived genes in FXD. Altered expression of those TE-derived genes changes purine metabolisms, promotes low O_2_ tension, interferes with immune responses, and induces inflammation. This explains why XO activity is elevated in FXD (7) (Figure 5C, ‘Present’). Previously, we found that the DNA alkylating environment is sustained in FXD by impairment of alkylation damage signaling through disruption of MPTAC (7). MPTAC regulates splicing (12), which is misregulated in FXD. These factors may lead to immune disorders in FXD (6). The outcome of gained A T to C G substitution and increased CG dinucleotides in FXD resembles successful latent viral gene expression and is likely to favor sustaining latent viral infections (Figure 5C, ‘Past’). As host monkey DNA sequences have been found in mutant SV40 virus genome (57), exchanging of host-specific DNA sequence with viral genes for viral benefit has been suggested (58). Hence, we propose that pathogenesis of FXD may be modulated by genes from past virus infections that are latently expressed as activated transposons (Figure 5C).

## Supplementary Material

ugae015_Supplemental_Files

## Data Availability

Original data associated with this manuscript is available from NCBI GEO with GSE159244 and GSE264091 and the Stowers Original Data Repository at http://www.stowers.org/research/publications/libpb-2511.
