# Supplementary material for: C G composition in transposon-derived genes is increased in FXD with perturbed immune system
Source: NAR Mol Med. 2024 Oct 10;1(4):ugae015. doi: 10.1093/narmme/ugae015 (PMC11500580; doi:10.1093/narmme/ugae015)

SUPPLEMENTARY DATA

Supplementary Figures and Table Legends

Supplementary Figure 1. Translation of viral response genes is up-regulated by XDH-knockdown.

(A) Cytoplasmic extracts from MOCS2- and XDH-knockdown and control HEK293 cells were examined by Western blots. eIF4E was used as the loading control. **(B)** Polysome profiles of MOCS2- and XDH-knockdown and control HEK293 cells. The profile of each knockdown is displayed in the right three panels. **(C)** Area-proportional Venn diagrams indicating the numbers of genes with significantly changed transcript levels (adjusted p-value < 0.05 and fold change > 2) in the input (In) for polysome fractionation, or in polysomes (po), in MOCS2- or XDH-knockdown cells compared to controls (n=2 or 3) (1). ContIn, total RNA from input in control cells; MOCS2KDIn/ XDHKDIn, total RNA from input in MOCS2- or XDH-KD; ContPo, total RNA from polysomes in control cells; MOCS2KDPo/ XDHPo, total RNA from polysomes in MOCS2- or XDH-KD. **(D and E)** Dot plots display GO analysis clustered by biological process (D) or molecular functions (E) for significantly increased (D) or decreased (E) transcripts in polysomes (≥3) from XDH-knockdown versus polysomes from control cells analyzed by RNA-seq data (Figure 1C). Representative genes are indicated.

Supplementary Figure 2. Substitutions of A T to C G in TE-derived genes are more abundant in female FXD compared to healthy female donors.

Heatmap with rows as each group of genes corresponding to Figure 2B and columns as samples display Z-score of ratio of A T>C G / C G>A T in averaged sum (n=3) of the base substitutions shown in Figure 2C.

Supplementary Figure 3. Counts of 5’-CG-3’ dinucleotides in TE-derived genes in male FXD groups and male healthy donor groups.

Box plots display the count of CG di-nucleotides normalized to length of the gene in 1000bp using the ATGC consensus in each group of TE-derived genes corresponding to the groups of Venn diagram in Figure 2B. Sample names and repeats are indicated at the bottom. The count in each repeat (n=3) is indicated. ALL*: ALL-lymphosarcoma (Figure 3A). Counterpart groups due to comparison between male healthy donors and male FXD (Figure 2B) are indicated.

Supplementary Figure 4. Counts of 5'-CG-3' dinucleotides in TE-derived genes in female FXD groups and female healthy donor groups.

Box plots display the count of CG di-nucleotides normalized to length of the gene in 1000bp using the ATGC consensus in each group of TE-derived genes corresponding to the groups of Venn diagram in Figure 2B. Sample names and repeats are indicated at the bottom. The count in each repeat (n=3) is indicated. ALL*: ALL-lymphosarcoma (Figure 3B). Counterpart groups due to comparison between female healthy donors and female FXD (Figure 2B) are indicated.

Supplementary Figure 5. Counts of 5'-GC-3' dinucleotides in TE-derived genes in male FXD groups and male healthy donor groups.

Box plots display the count of GC di-nucleotides normalized to length of the gene in 1000 bp using the ATGC consensus in each group of TE-derived genes corresponding to the groups of Venn diagram in Figure 2B. Sample names and repeats are indicated at the bottom. The count in each repeat (n=3) is indicated. ALL*: ALL-lymphosarcoma. Counterpart groups due to comparison between male healthy donors and male FXD (Figure 2B) are indicated.

Supplementary Figure 6. Counts of 5'-GC-3' dinucleotides in TE-derived genes in female FXD groups and female healthy donor groups.

Box plots display the count of GC di-nucleotides normalized to length of the gene in 1000 bp using the ATGC consensus in each group of TE-derived genes corresponding to the

groups of Venn diagram in Figure 2B. Sample names and repeats are indicated at the bottom. The count in each repeat (n=3) is indicated. ALL*: ALL-lymphosarcoma. Counterpart groups due to comparison between female healthy donors and female FXD (Figure 2B) are indicated.

Supplementary Figure 7. CpG islands in TE-derived genes with/without changed expression in FXD groups.

(A) Area-proportional Venn diagrams display the numbers of CpG-containing genes in TE-derived genes in each group of TE-derived genes corresponding to the groups of Venn diagram in Figure 2B. **(B and C)** Line plots display locations of these CpG islands in the gene body ± 2kb flanking regions in each group of TE-derived genes in (A).

Supplementary Figure 8. Citrate synthase activities were altered in FXD.

The production of Coenzyme A via citrate synthase in mitochondrial extracts from indicated LCLs was monitored over time by measuring the absorbance of citrate synthase developing reagent at 412 nm.

Supplementary Figure 9. Splicing of *HLA* genes was changed in FXD.

(A) Sashimi plots (2) display that the splicing of *HLA-DMA* in TAS1 (male FXD) was changed vs. male healthy donors (TAS10, 23, 41, and 42) (Figure 4C). Genomic reads were converted into read densities (measured in RPKM). Junction reads are plotted as arcs. Width of arcs is determined by the number of reads aligned to the junctions ranging the exons. The junction depth in text number for each arc is displayed (B). **(B)** The splicing of *HLA-A* in TAS2 and TAS19 (female FXD) was changed vs. female healthy donors (TAS11, 22, 30, and 43) (Figure 4C).

Supplementary Figure 10. Splicing of *ERAP2* was changed in male FXD.

Sashimi plots (2) display that the splicing of *ERAP2* in TAS1, 18, and 20 (male FXD) was changed vs. LCLs from male healthy donors (TAS10, 23, 41, and 42) (Figure 4D).

Genomic reads were converted into read densities (measured in RPKM). Junction reads are plotted as arcs. Width of arcs is determined by the number of reads aligned to the junctions ranging the exons. The junction depth in text number for each arc is displayed.

Supplementary Table 1. Genes with significantly decreased or increased transcript levels in polysomes from MOCS2- and XDH-knockdown cells.

Genes found in GO terms clustered by biological process (BP) or molecular functions (MF) for significantly reduced/increased transcripts (RPKM>1) in polysomes (≥3) from MOCS2- and/or XDH-knockdown versus polysomes from control cells analyzed by RNA-seq data are listed (Figures 1C, 1D, and 1E; Supplementary Figures S1D and S1E).

Supplementary Table 2. List of LCLs.

Overview of LCLs used in this study. M, male; F, female; y, years old; mo, months. Diagnosis/ descriptions is from the official website of Coriell Institute for Medical Research. Female LCLs: TAS2, TAS21 and TAS29 samples may actually be FXS premutation carriers. FMR1 protein expression was observed in TAS2 and TAS21 but not in TAS19 and TAS29 (3).

Supplementary Table 3. TE-derived genes with changed splicing in FXS cells versus healthy donor cells.

TE-derived genes with changed splicing (Figure 2B) found in GO terms clustered by biological process (BP) in male or female FXS LCLs compared to male or female healthy donors LCLs, respectively, based on RNA-seq are listed (p-val.<0.05) (Figures 4A and 4B; Supplementary Figures S9 and S10). ALL*: ALL-lymphosarcoma.

Supplementary Table 4. TE-derived genes with changed splicing in FXS cells versus healthy donor cells.

TE-derived genes with differential splicing between FXD and healthy donors in each gender analyzed by rMAT (v4.1.1) are listed (filtered at FDR 5 %) (2) (Figures 4A and 4B; Supplementary Figures S9 and S10). SE: Skipped exon, MXE: Mutually exclusive exon.

Supplementary Table 5. TE-derived genes with changed splicing or significantly changed transcript levels in FXS cells versus female healthy donor cells.

TE-derived genes with changed splicing (at 5 % FDR) or transcript levels (p-values corrected with the FDR method. Genes at 5 % FDR with a log2(fold change) > 1 for up-regulated genes and log2(fold change) < -1 for down-regulated were counted as differentially expressed genes) in FXS LCLs compared to healthy donors LCLs sorted with gene locations based on RNA-seq are listed. (Figures 2B, 4A, and 4B).

Supplementary Table 6. TE-derived genes with significantly changed transcript levels in male FXS cells versus male healthy donor cells.

TE-derived genes with significantly changed transcript levels (Adj p-val.<0.05) (Figure 2B) found in GO terms clustered by molecular functions (MF) or biological process (BP) in male FXS LCLs compared to healthy male or female donors LCLs, respectively, based on RNA-seq are listed (Figures 4E and 4F).

Supplementary References

1. Suganuma, T., Swanson, S.K., Gogol, M., Garrett, T.J., Florens, L. and Workman, J.L. (2022) MOCS2 links nucleotide metabolism to nucleoli function. *Journal of molecular cell biology*, **13**, 838-840.
2. Katz, Y., Wang, E.T., Silterra, J., Schwartz, S., Wong, B., Thorvaldsdottir, H., Robinson, J.T., Mesirov, J.P., Airoldi, E.M. and Burge, C.B. (2015) Quantitative visualization of alternative exon expression from RNA-seq data. *Bioinformatics*, **31**, 2400-2402.
3. Suganuma, T. and Workman, J.L. (2022) MPTAC links alkylation damage signaling to sterol biosynthesis. *Redox biology*, **51**, 102270.

Suganuma Figure S1

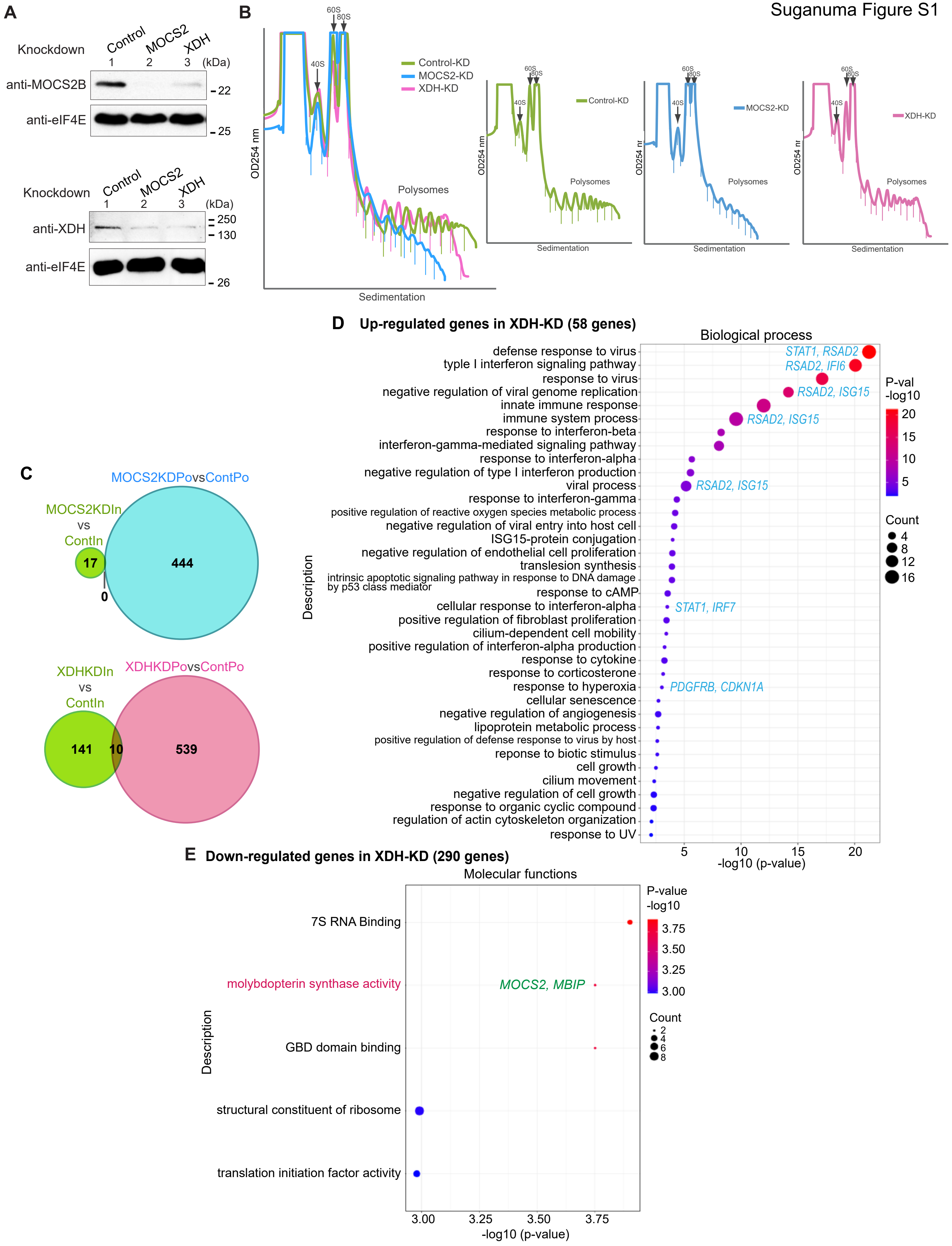

Suganuma Figure S2

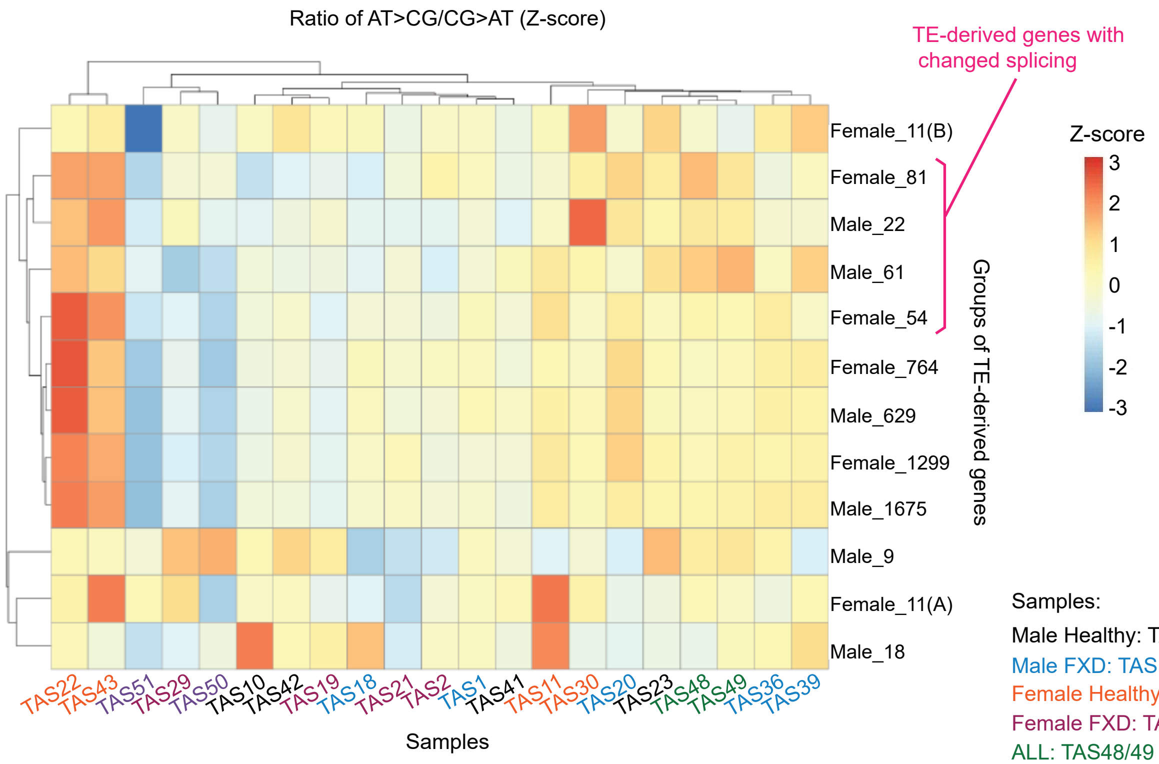

Samples:

Male Healthy: TAS10/23/41/42

Male FXD: TAS1/18/20/36/39

Female Healthy: TAS11/22/30/43

Female FXD: TAS2/19/21/29

ALL: TAS48/49

ALL-Lymphosarcoma: TAS50/51

Male groups

Suganuma Figure S3

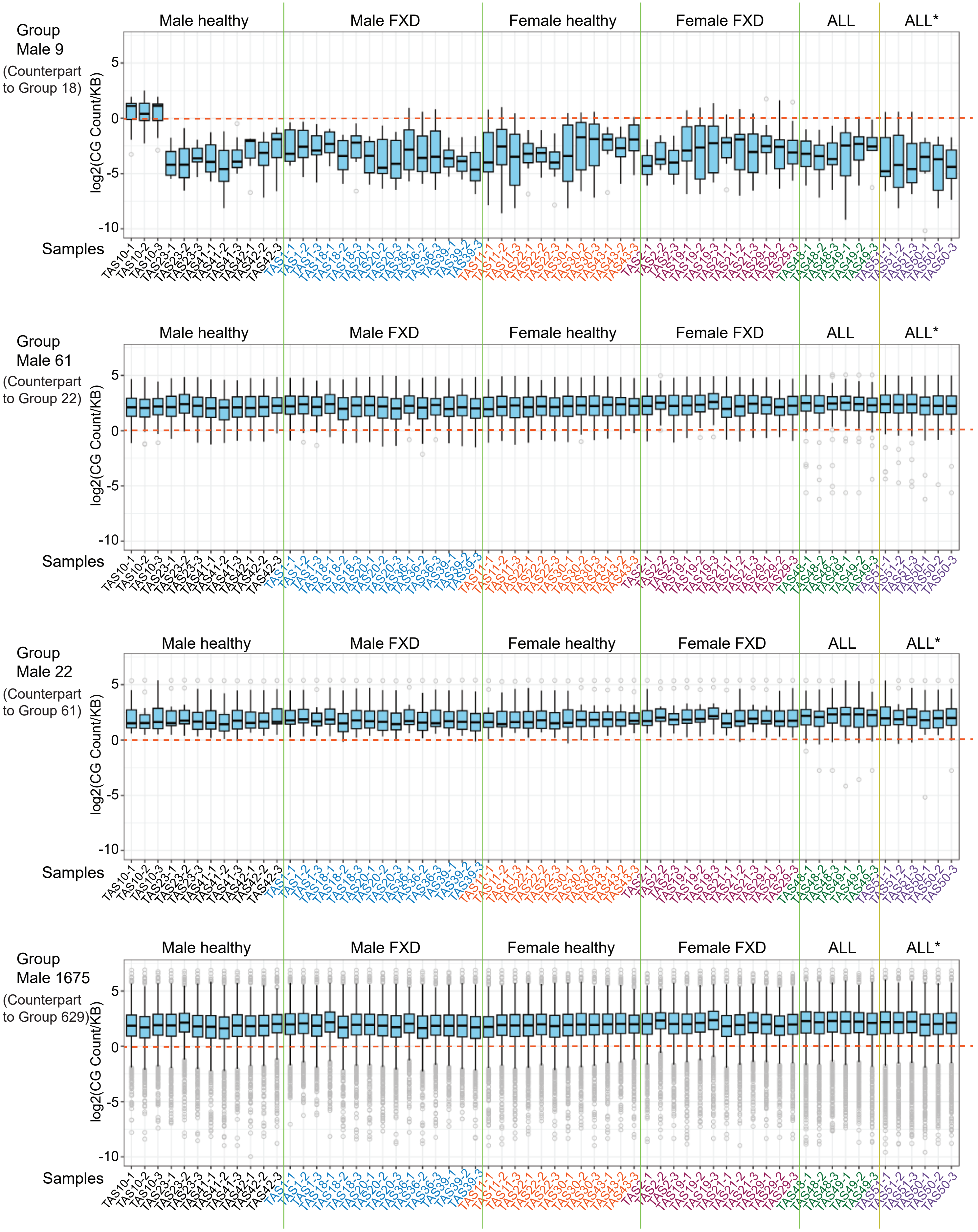

Suganuma Figure S4

Female groups

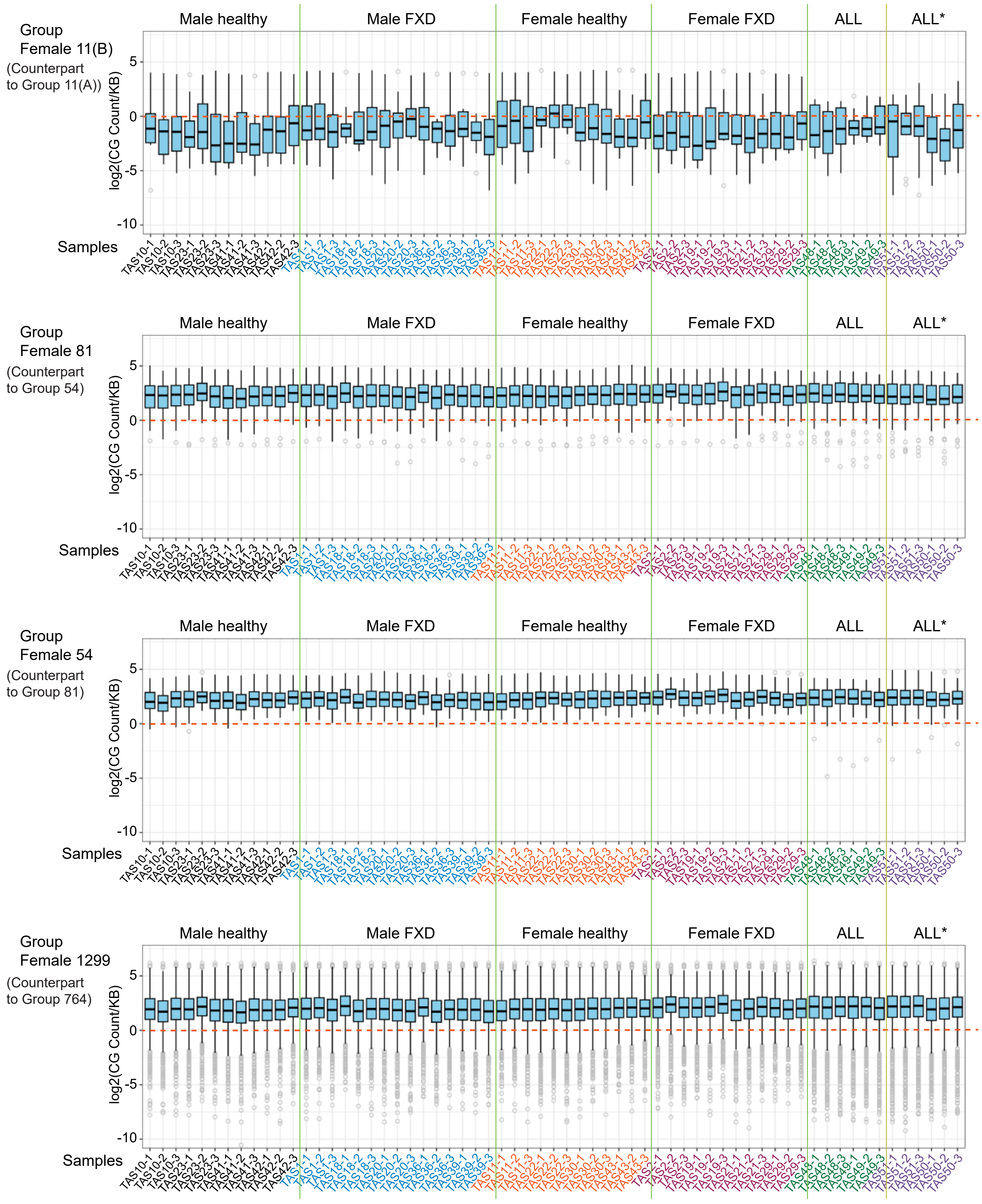

Male groups

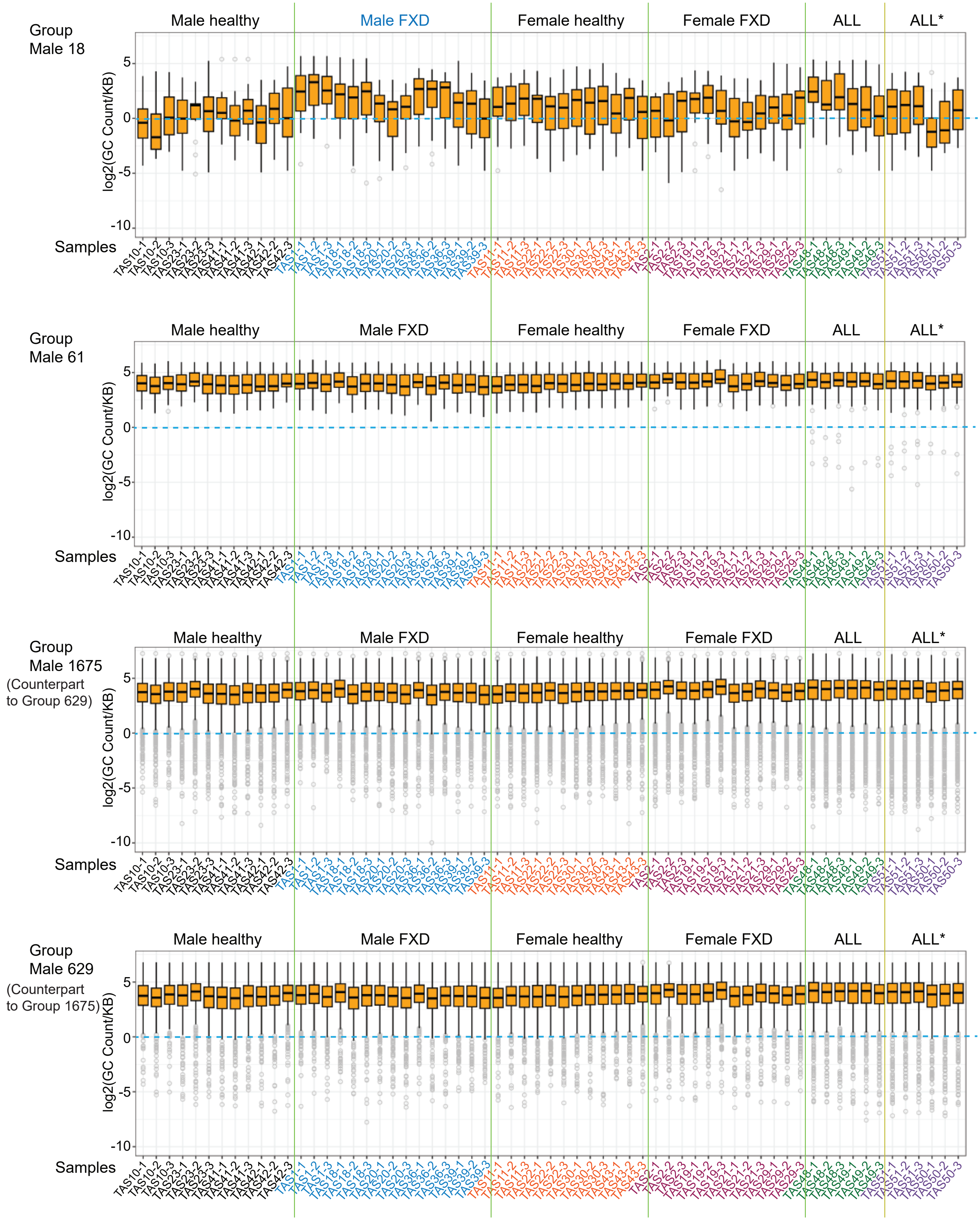
Group Male 18
Group Male 61
Group Male 1675
(Counterpart to Group 629)
Group Male 629
(Counterpart to Group 1675)
Male healthy
Male FXD
Female healthy
Female FXD
ALL
ALL*
log2(GC Count/KB)
5
0
-5
-10
Samples
TAS10-1
TAS10-2
TAS10-3
TAS23-1
TAS23-2
TAS23-3
TAS41-1
TAS41-2
TAS41-3
TAS42-1
TAS42-2
TAS42-3
TAS1-1
TAS1-2
TAS1-3
TAS18-1
TAS18-2
TAS18-3
TAS20-1
TAS20-2
TAS20-3
TAS36-1
TAS36-2
TAS36-3
TAS39-1
TAS39-2
TAS39-3
TAS11-1
TAS11-2
TAS11-3
TAS22-1
TAS22-2
TAS22-3
TAS30-1
TAS30-2
TAS30-3
TAS43-1
TAS43-2
TAS43-3
TAS2-1
TAS2-2
TAS2-3
TAS19-1
TAS19-2
TAS19-3
TAS21-1
TAS21-2
TAS21-3
TAS29-1
TAS29-2
TAS29-3
TAS48-1
TAS48-2
TAS48-3
TAS49-1
TAS49-2
TAS49-3
TAS51-1
TAS51-2
TAS51-3
TAS50-1
TAS50-2
TAS50-3

Female groups

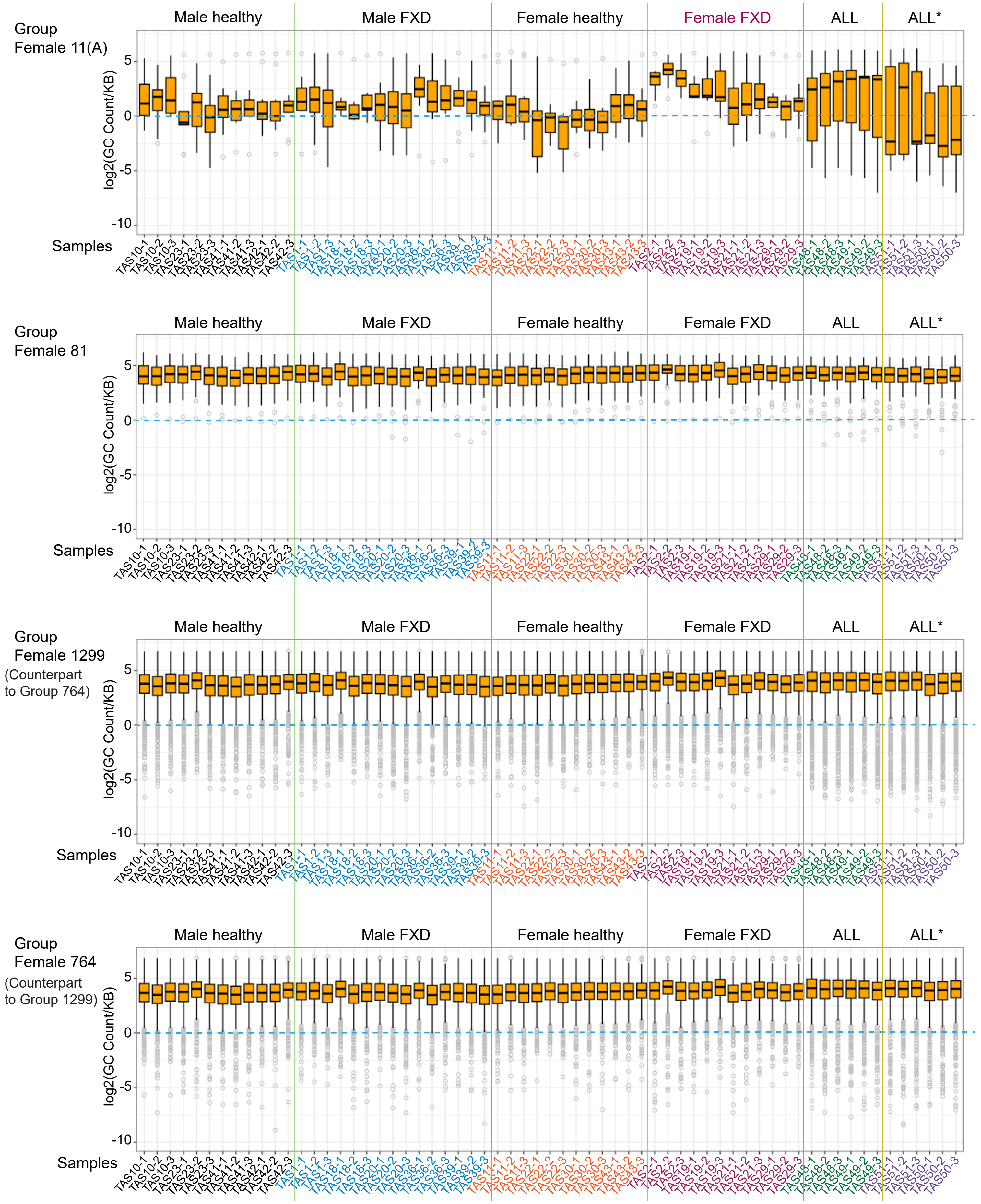

Suganuma Figure S7

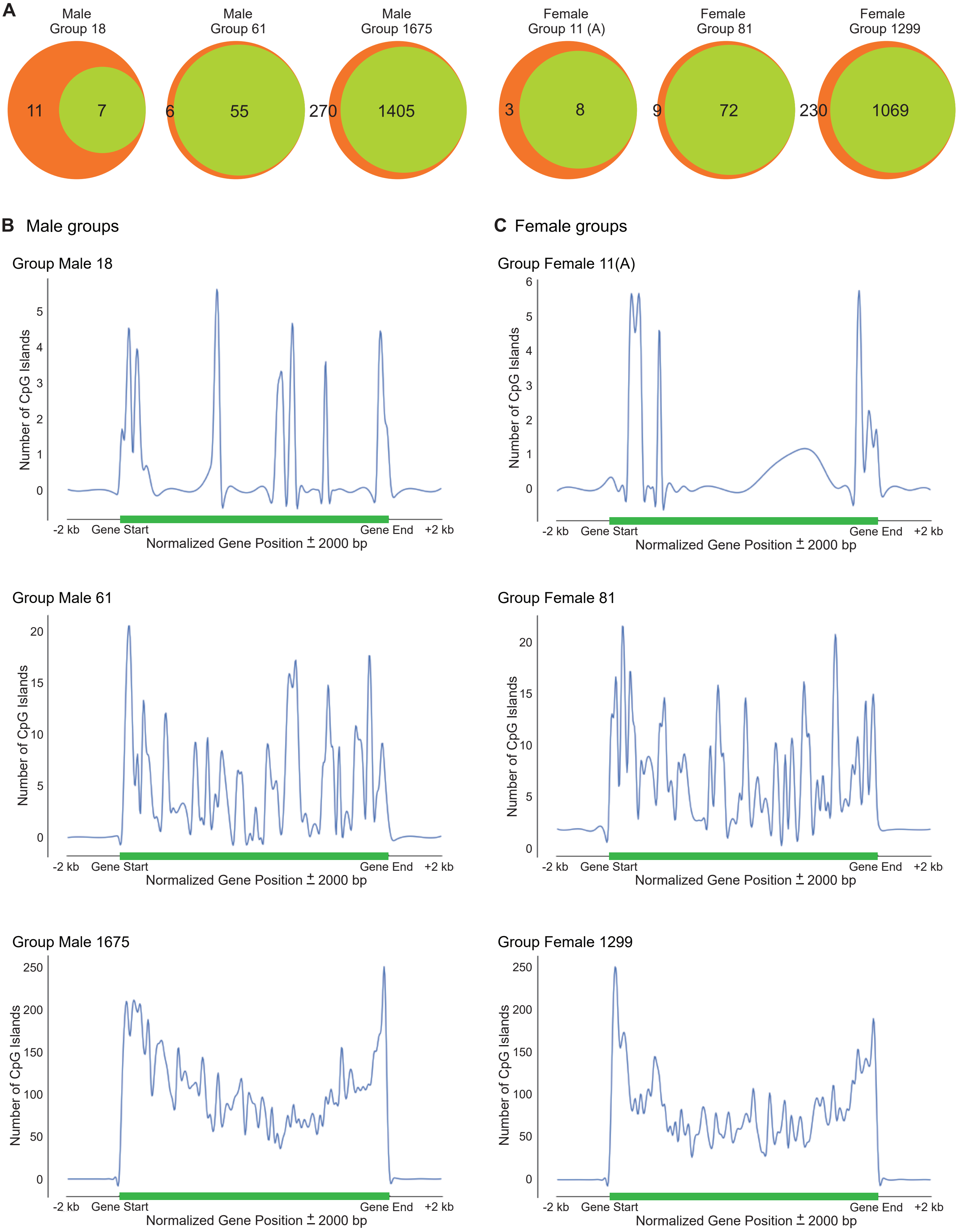

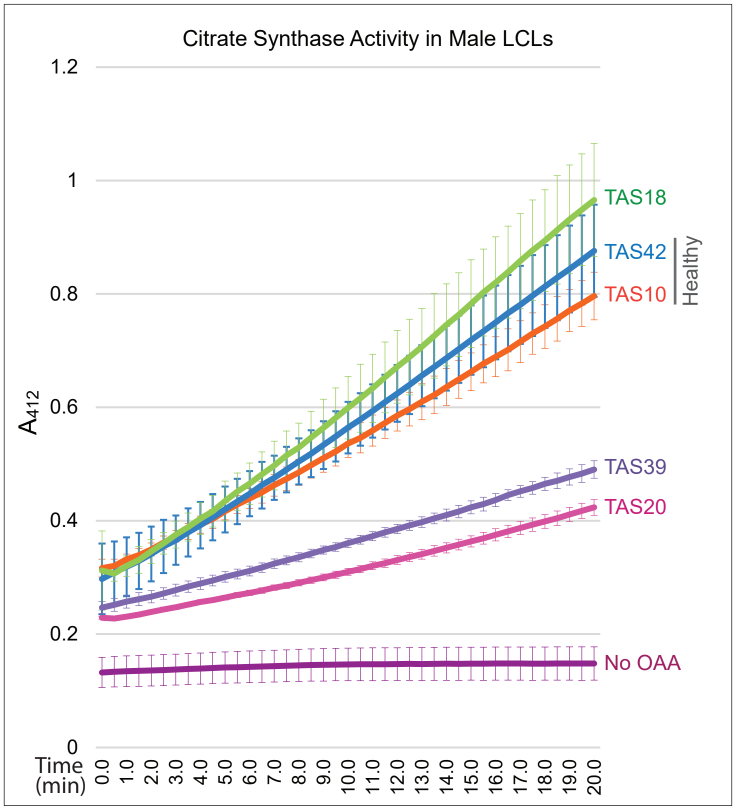

Citrate Synthase Activity in Male LCLs
1.2
1
0.8
0.6
0.4
0.2
0
A_{412}
Time (min)
0.0 1.0 2.0 3.0 4.0 5.0 6.0 7.0 8.0 9.0 10.0 11.0 12.0 13.0 14.0 15.0 16.0 17.0 18.0 19.0 20.0
TAS18
TAS42
TAS10
Healthy
TAS39
TAS20
No OAA

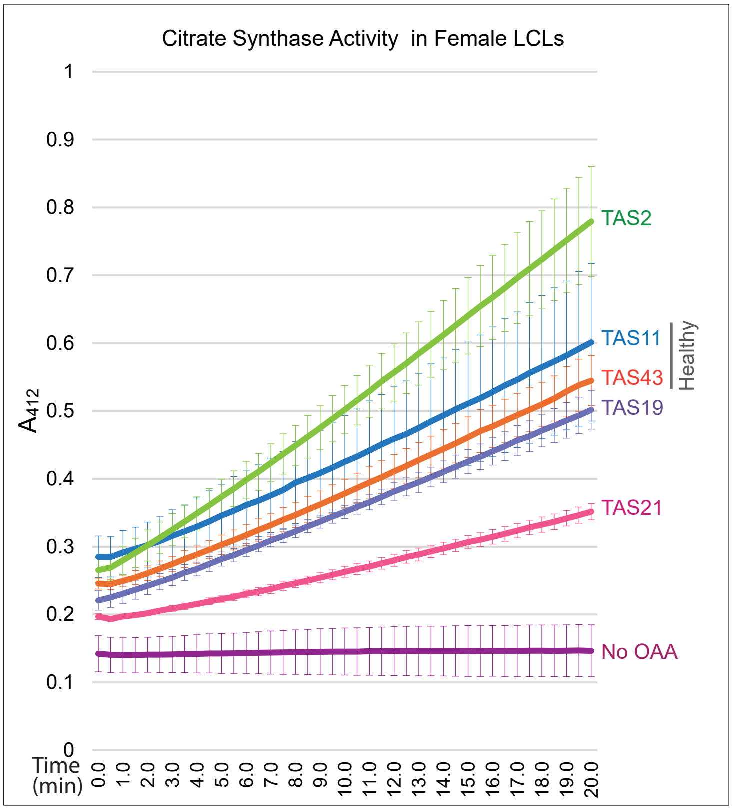

Citrate Synthase Activity in Female LCLs
1
0.9
0.8
0.7
0.6
0.5
0.4
0.3
0.2
0.1
0
A_{412}
Time (min)
0.0 1.0 2.0 3.0 4.0 5.0 6.0 7.0 8.0 9.0 10.0 11.0 12.0 13.0 14.0 15.0 16.0 17.0 18.0 19.0 20.0
TAS2
TAS11
TAS43
TAS19
Healthy
TAS21
No OAA

A

HLA-DMA

chr6_32950519_32950803_-@ch6_32949611_32950803_
-@ch6_32949611_32949787_-

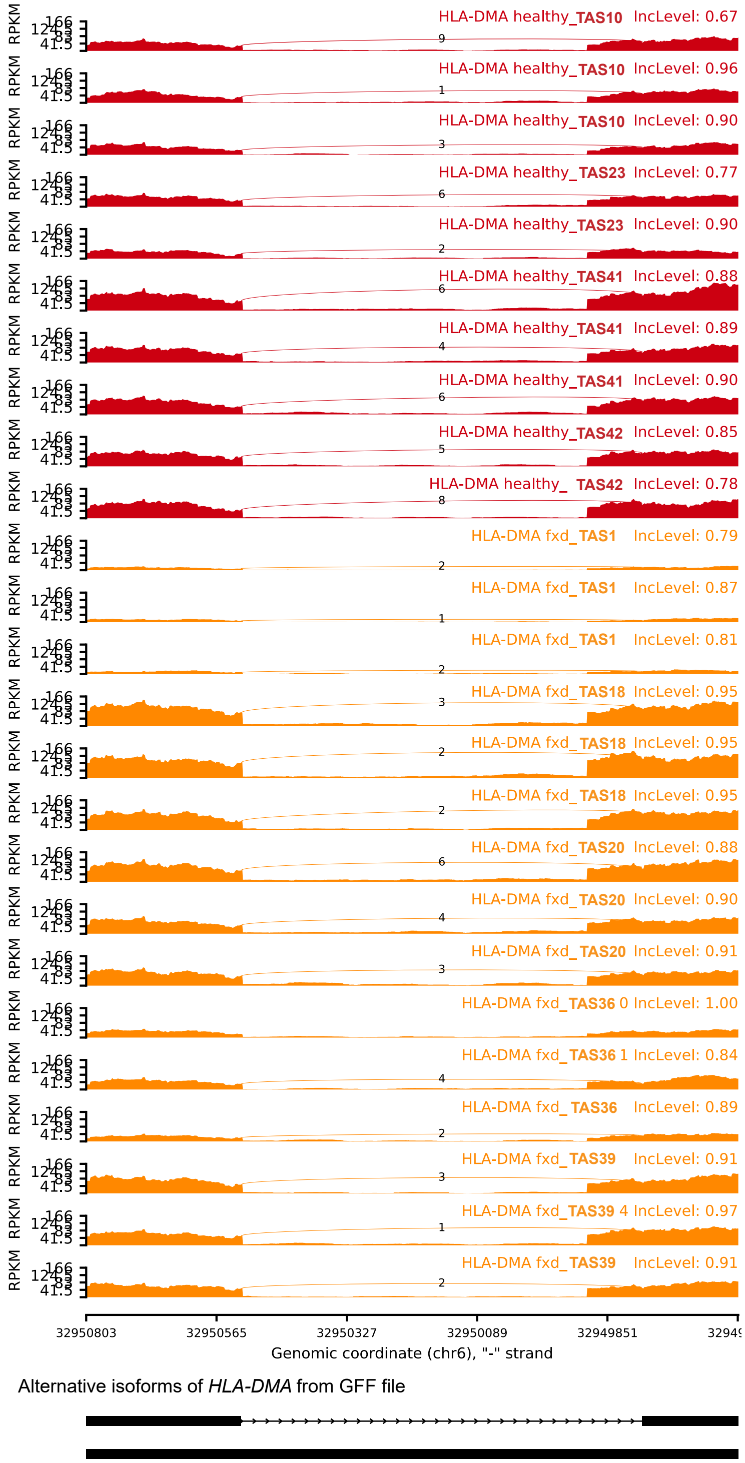

B

HLA-A

chr6_29942757_29943026_+@chr6_29943268_29943543_
+@29944122_29944397_+@_29945059_

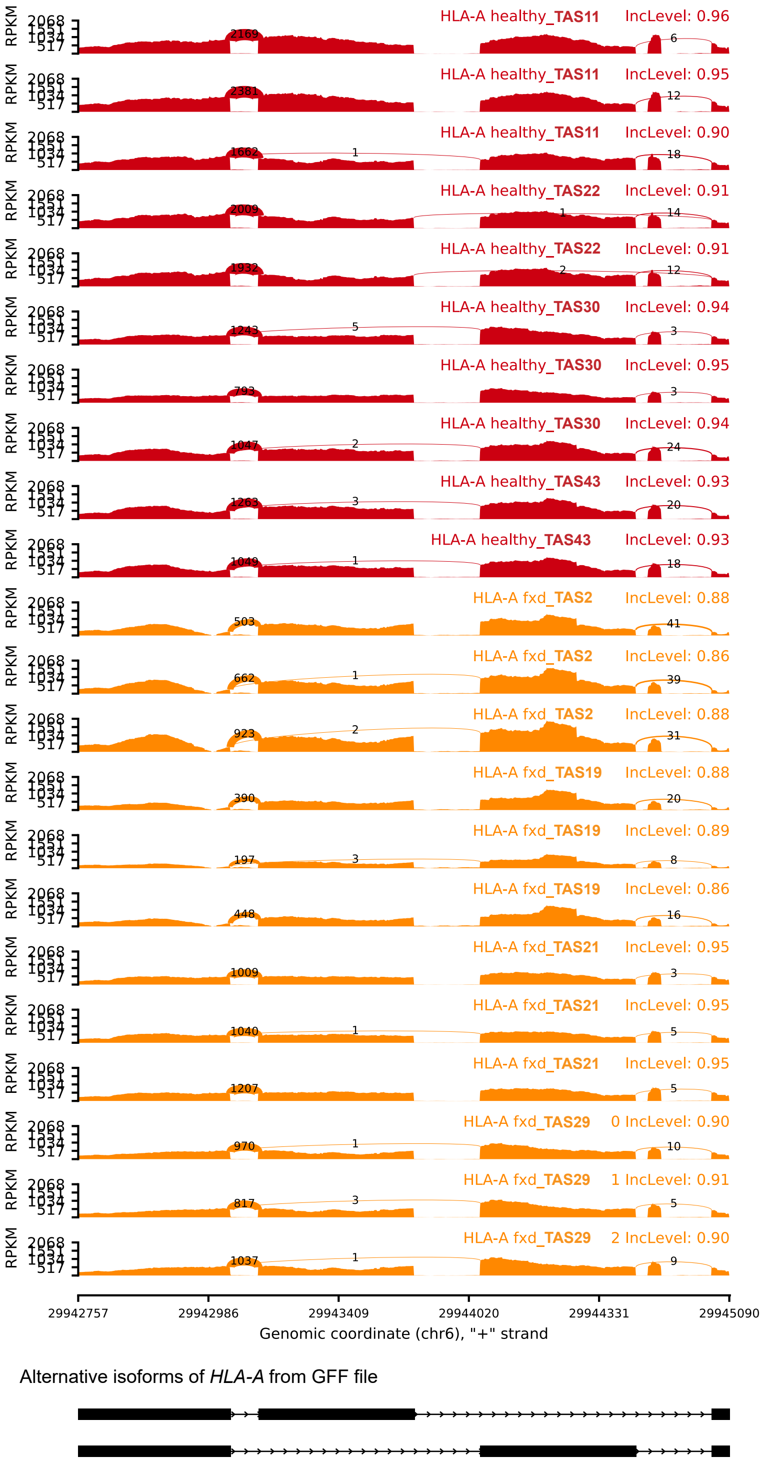

Suganuma Figure S10

ERAP2

_96896373_96896504_+@chr5_96896732_96896863_
+@chr5_96900121_96900189_+@chr5_96901506_

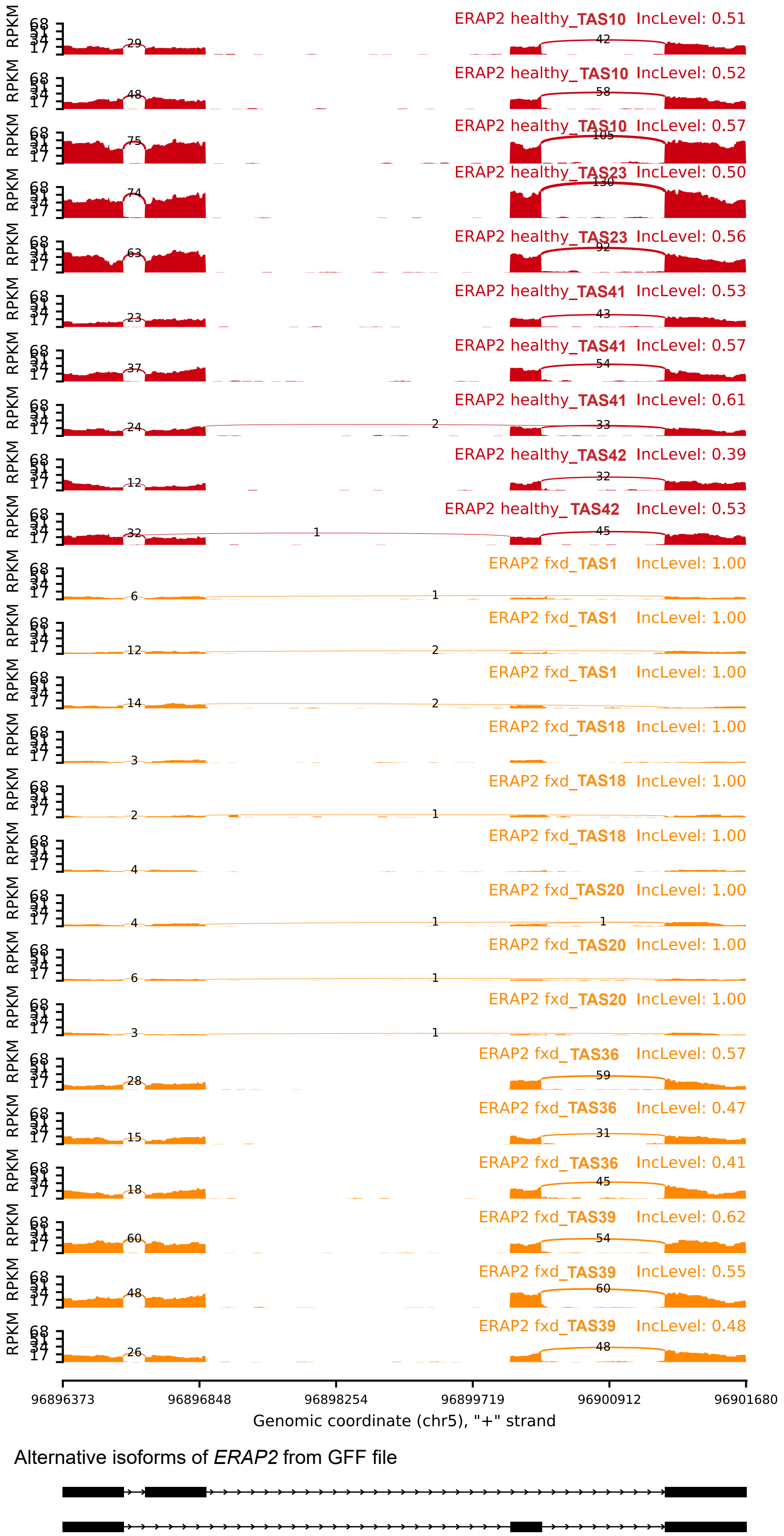

Supplement: ugae015_Supplemental_Files [file ugae015_Supplemental_Files.zip › Suganuma(2)_SupplementalDataFigs1-10_Revision_NARMME-2024-020.R1_clean.pdf]
